# Comment on García-Erce et al. Iron Deficiency and Oral Treatments: Limitations, Pharmacokinetics, and the Role of Iron Protein Succinylate in Clinical Practice. *J. Clin. Med.* 2026, *15*, 3691

**DOI:** 10.3390/jcm15176767

**Published:** 2026-08-31

**Authors:** Germano Tarantino, Maria Sole Rossato, Camilla Vullo, Elisa Brilli

**Affiliations:** PharmaNutra S.p.A., 56122 Pisa, Italy

The purpose of this letter is to comment on the review titled “Iron Deficiency and Oral Treatments: Limitations, Pharmacokinetics, and the Role of Iron Protein Succinylate in Clinical Practice” by García-Erce et al. (2026) [1].

We read it with great interest, since it addresses the potential advantages of iron protein succinylate (IPS), a ferric complex bound to succinylated casein, over traditional iron salts in the context of iron deficiency management. We would like to commend the authors for providing a clear and comprehensive overview of the physiology of iron absorption, while thoughtfully outlining the limitations of conventional oral iron therapies. We particularly appreciate the discussion placing IPS in relation to Sucrosomial^®^ Iron (SI), which adds valuable perspective to the current therapeutic landscape. However, we believe that further clarification on specific aspects may help to strengthen the overall interpretation.

SI is a formulation of ferric pyrophosphate conveyed with the phospholipids and sucrose esters of a fatty acid (sucrester) matrix [2]. This formulation is significantly distinct from other products containing other sources of iron (e.g., microencapsulated or micronized iron), as demonstrated in numerous scientific publications [2].

Reading the manuscript by García-Erce et al. (2026), we identified three main inaccuracies concerning SI: the first two in section “Comparative Benefits of IPS in Clinical Studies”, paragraph “Optimized Hematologic Efficacy and Bioavailability with Shorter Treatment”, the latter one regarding Table 3 “Key differences between IPS and conventional oral iron formulations (ferrous and ferric)”:

1. On page 10, the statement “…suggesting that, while sucrosomial iron can correct anemia, it may not fully restore iron stores” appears to generalize a finding (i.e., lack of ferritin repletion) observed in a single study [3], presenting it as an intrinsic limitation of SI. However, drawing broad conclusions about a compound’s efficacy requires consideration of the entirety of the available scientific literature, rather than reliance on isolated evidence. Indeed, several published studies, conducted over a short period of time (three months) have reported a significant increase in ferritin levels following treatment with SI, supporting its ability to replenish iron stores over time. Examples of these full-text publications are reported in Table 1. Notably, the restoration of iron stores appears to become more pronounced with a longer treatment duration. This is illustrated by a study presented by Dr. Ioannis Griveas at the 5th International Multidisciplinary Course on Iron Anemia in 2017 [4], in which 18 months of SI treatment in patients with chronic kidney disease (CKD) led to a substantial increase in ferritin levels, from 42.73 ± 24.47 ng/mL at baseline to 98.89 ± 126.99 ng/mL at follow-up. These findings suggest that prolonged SI therapy may contribute to a progressive replenishment of iron stores beyond the improvements observed during shorter treatment periods. However, iron store replenishment cannot be assessed solely on the basis of ferritin levels, as ferritin is an acute-phase reactant and therefore does not always accurately reflect body iron stores, especially during chronic inflammation. Nevertheless, considering that SI absorption is hepcidin-independent and therefore remains efficient even in the presence of inflammation, it can be hypothesized that the observed increase in ferritin more closely reflects an actual improvement in iron stores.

2. The statement “sucrosomial iron is not recognized by the European Medicines Agency or national medicines agencies as a medication, but it is considered a nutritional supplement. Consequently, the evidence for its benefit and safety in treating ID has not undergone the rigorous evaluation of these agencies”, reported on page 10 may be misleading. Indeed, while it is correct that SI is classified as a nutritional supplement rather than a pharmaceutical product, a substantial body of scientific evidence demonstrates its efficacy in rapidly and effectively improving hemoglobin levels in anemic iron-deficient patients across different clinical settings [2]. Furthermore, ID itself represents a deficiency in a mineral nutrient (i.e., iron) that can be recovered through proper nutritional support or food supplements, that moreover are safe under recommended conditions of use. Unlike medicinal products, the safety assessment is based on known ingredient profiles and established intake levels. In addition, compared to certain medicinal iron formulations approved, for example, by the Italian Medicines Agency (AIFA) (e.g., FERPLEX, containing IPS), SI does not include excipients typically contained in drugs that may be associated with tolerability concerns, such as propylene glycol (E1520), methyl parahydroxybenzoate sodium salt (E219), propyl parahydroxybenzoate sodium salt (E217), sodium saccharin, and sodium hydroxide. Additionally, SI is marketed in 79 countries in Europe, North America, South America and Asia, with millions of doses sold and without any communication of any health hazard to national health authorities.
3. Table 3 of García-Erce et al. [1] summarizes the scientific evidence supporting the advantages of IPS over conventional oral iron products, namely simple iron salts such as ferrous sulfate and ferrous gluconate. These compounds are absorbed through a DMT1/ferroportin-dependent pathway, a mechanism that not only constrains iron bioavailability, but is also associated with an increased amount of unabsorbed iron in the intestinal lumen, contributing to gastrointestinal side effects through the generation of Fenton’s reactions [9]. In this context, SI cannot be appropriately classified together with simple iron salts within the same comparative framework. Indeed, SI, owing to its unique and patented formulation, is characterized by marked gastro-resistance properties and a unique and unconventional absorption mechanism. The distinctive gastro-resistant profile is conferred by the sucrester matrix [10], which hides and protects the iron as it passes through the gastric environment. Subsequently, the absorption mechanism is independent of the DMT1/ferroportin axis, and therefore also hepcidin-independent. SI is indeed absorbed through transcellular and paracellular pathways, as well as via M cells, allowing it to bypass the absorption limitations of traditional iron salts and resulting in enhanced bioavailability. In addition, Table 3 reports: “sucrosomial iron does not replenish iron stores”, a statement that, beyond its inherent inaccuracy as previously discussed, is supported by a study that evaluated the effect of IPS versus ferrous sulfate on ferritin levels [11]. However, SI was not included in this study. Therefore, the reported statement is not supported by the cited reference (Ref. 61 in the paper).

To promote clarity, completeness, and scientific rigor for the benefit of the reader, we kindly suggest that the authors:

1. Revise the statements regarding the inability of SI to restore iron stores, both in the main text and in Table 3, avoiding generalizations based on single-source evidence.
2. Clarify that SI is classified as a food supplement and is considered safe under recommended conditions of use. Furthermore, a substantial and growing body of clinical evidence supports its efficacy across different clinical settings.
3. Remove or reposition SI from Table 3 as it does not belong to the class of simple iron salt formulations.
4. Correct the term “sucrosomial iron” to “Sucrosomial^®^ Iron” throughout the whole manuscript, acknowledging it as a registered trademark and specifying that the patented technology is exclusively licensed by PharmaNutra S.p.A. (Italy) (Patent No. PCT/IB2013/001659).

Not addressing these suggestions may lead to misleading interpretations and raise concerns regarding scientific rigor, as well as to potential reputational implications for SI. Furthermore, we encourage the authors to avoid drawing conclusions from single references and to ensure that cited sources accurately support the statements made, as reliance on limited or mismatched evidence may compromise the transparency and robustness of the scientific discussion.

## Figures and Tables

**Table 1:** Experimental evidence showing significantly increased serum ferritin levels following treatment with SI.

Reference	Clinical Setting	Treatment	Duration	Endpoint Examination	Serum Ferritin		*p*-Value
					**Baseline Mean (SD)**	**Endpoint Mean (SD)**	
Alexiadou et al., 2024 [5]	Pediatric patients with IDA or IDWA	SI 1.4 mg/day	3 months	3 months	6.9 ng/mL (3.5 ng/mL)	16.5 ng/mL (11.3 ng/mL)	<0.001 vs. baseline
					**Control Endpoint Increment**	**Treatment Endpoint** **Increment**	
Karavidas et al., 2021 [6]	HFrEF patients with ID	SI 28 mg/day vs. Control (no treatment)	3 months	3 months	−0.05 ng/mL	+28.34 ng/mL	<0.001 vs. control
				6 months	−0.91 ng/mL	+39.89 ng/mL	<0.001 vs. control
					**Control Endpoint** **Mean (SD)**	**Treatment Endpoint Mean (SD)**	
Parisi et al., 2016 [7]	Non-anemic pregnant women	SI 14 mg/day vs. Control (no treatment)	11–13th gestational week to 6 weeks post-partum	6 weeks post-partum	31.3 ng/mL (15.4 ng/mL)	40.8 ng/mL (32.5 ng/mL)	<0.02 vs. control
		SI 28 mg/day vs. Control (no treatment)				49.8 ng/mL (12.6 ng/mL)	<0.001 vs. control
					**Baseline Median (IQR)**	**Endpoint Median (IQR)**	
Torreiter et al., 2022 [8]	PrW	SI 30 mg/day	90 days	90–120 days	8.3 ng/mL (6.8, 10.3 ng/mL)	14.5 ng/mL (11.1, 17.1 ng/mL)	<0.001 vs. baseline
	PoW				10.3 ng/mL (6.3, 16.2 ng/mL)	18.4 ng/mL (12.5, 24.5 ng/mL)	0.005 vs. baseline
	Men				10.1 ng/mL (8.2, 13.8 ng/mL)	16.0 ng/mL (8.2, 18.8 ng/mL)	0.035 vs. baseline
	PoW and men				10.3 ng/mL (7.3, 15.0 ng/mL)	16.9 ng/mL (12.3, 22.6 ng/mL)	<0.001 vs. baseline

**IDA = iron deficiency anemia; IDWA = iron deficiency without anemia; SI = Sucrosomial^®^ Iron;** HFrEF = heart failure patients with reduced ejection fraction; ID = iron deficiency; PrW = premenopausal women; PoW = postmenopausal women.

## Data Availability

No new data were created or analyzed in this study. Data sharing is not applicable to this article.
